# The Model of Aging Acceleration Network Reveals the Correlation of Alzheimer's Disease and Aging at System Level

**DOI:** 10.1155/2019/4273108

**Published:** 2019-07-14

**Authors:** Mengyu Zhou, Xiaoqiong Xia, Hao Yan, Sijia Li, Shiyu Bian, Xianzheng Sha, Yin Wang

**Affiliations:** ^1^Department of Biomedical Engineering, School of Fundamental Sciences, China Medical University, Shenyang 110122, Liaoning Province, China; ^2^China Medical University-The Queen's University of Belfast Joint College, China Medical University, Shenyang 110122, Liaoning Province, China; ^3^Tumor Etiology and Screening Department of Cancer Institute and General Surgery, The First Affiliated Hospital of China Medical University, 155# North Nanjing Street, Heping District, Shenyang 110001, Liaoning Province, China

## Abstract

As the incidence of senile dementia continues to increase, researches on Alzheimer's disease (AD) have become more and more important. Several studies have reported that there is a close relationship between AD and aging. Some researchers even pointed out that if we wanted to understand AD in depth, mechanisms of AD based on accelerated aging must be studied. Nowadays, machine learning techniques have been utilized to deal with large and complex profiles, thus playing an important role in disease researches (i.e., modelling biological systems, identifying key modules based on biological networks, and so on). Here, we developed an aging predictor and an AD predictor using machine learning techniques, respectively. Both aging and AD biomarkers were identified to provide insights into genes associated with AD. Besides, aging scores were calculated to reflect the aging process of brain tissues. As a result, the aging acceleration network and the aging-AD bipartite graph were constructed to delve into the relationship between AD and aging. Finally, a series of network and enrichment analyses were also conducted to gain further insights into the mechanisms of AD based on accelerated aging. In a word, our results indicated that aging may contribute to the development of AD by affecting the function of the immune system and the energy metabolism process, where the immune system may play a more prominent role in AD.

## 1. Introduction

It has been reported that the number of people who develop dementia is increasing rapidly—one case every 3 seconds [1]. In 2018, there were about 50 million people in the world affected by dementia, and the main form was Alzheimer's disease (AD). Actually, AD is a very common neurodegenerative disease characterized by the abnormal level of amyloid *β*-peptide (A*β*) and tau protein [2]. Alzheimer's disease can affect the patients' normal life and reduce the life quality. Moreover, the cost of the treatment is very expensive, resulting in an economic impact on the health care system [3]. With the growth of elderly people, it is not difficult to conclude that dementia will become a global problem. 

Obviously, AD has its distinct etiology and neuropathology. However, it has been proposed that if we wanted to have a comprehensive understanding of AD, we must study the relationship between aging and AD [4]. In fact, aging had a lot to do with AD. According to the Baltimore Longitudinal Study of Aging (BLSA), the incidence rate of AD increased visibly after the age of 60 [5]. That was to say aging brains were much susceptible to AD compared with young brains. Second, there was a phenomenon called normalcy-pathology homology. For example, in the frontotemporal region, cortical reductions were evident in elderly people, including those who were nondemented. Interestingly, these regions were also vulnerable to AD [6]. Third, AD patients and healthy elderly people had some common symptoms, such as the episodic memory decline [7, 8], brain atrophy [9–11], and the deposition of amyloid protein [12]. Moreover, the accelerated decline of brain volume has already been observed in mild cognitive impairment (MCI) compared with the normal aging, which was of great importance in understanding AD [10]. Therefore, we sought to study the relationship between aging and AD from the perspective of aging acceleration. We proposed a hypothesis that accelerated aging was one of the most important factors to promote the progression of AD by affecting some molecular mechanisms.

With the generation of large and complex datasets from biological experiments, methods to analyze these datasets became diverse. Machine learning is one of the most powerful tools to cope with the huge and complex datasets. It can learn the pattern of the given data and make predictions for new data [13]. More importantly, the strength of this approach is that it can sift through quantities of data to find predictive patterns. In addition, network biology can study the interactions between molecules and provide biological information based on the machine learning methods [14, 15].

**Figure 1:**
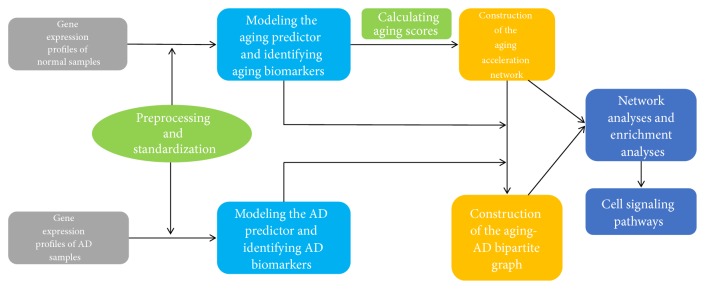
The workflow in this work.

In this paper, we utilized machine learning techniques to analyze the relationship between aging and AD and studied the mechanisms underlying them. First, we identified aging and AD related risk biomarkers, respectively. Second, we established an aging score comprising the aging biomarkers. Then the aging acceleration network was constructed based on the correlation between each pair of genes and aging scores. Finally, network analyses and enrichment analyses were also conducted to understand the relevant biological mechanisms. The workflow was shown in Figure 1. In summary, the association between aging and AD biomarkers was investigated by our computational pipeline.

## 2. Results and Discussion

### 2.1. Identification of Aging and AD Related Risk Biomarkers

**Figure 2:**
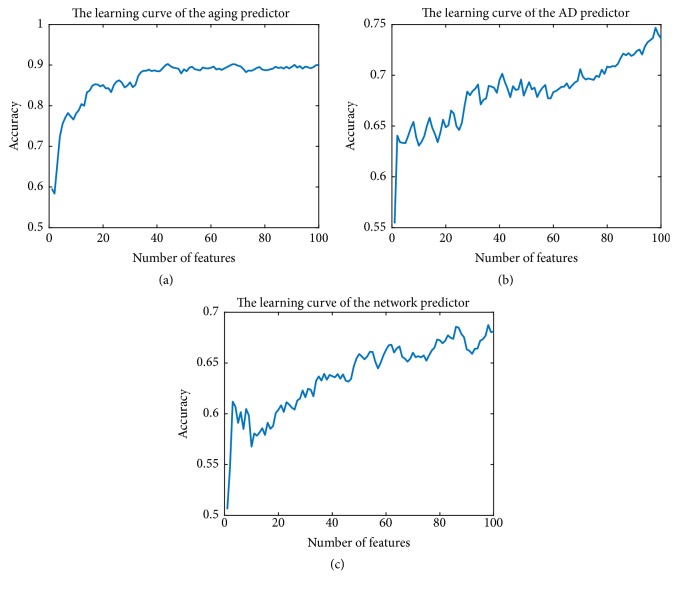
The learning curves of the three predictors. (a) The learning curve of the aging predictor; (b) the learning curve of the AD predictor; and (c) the learning curve of the network predictor.

**Figure 3:**
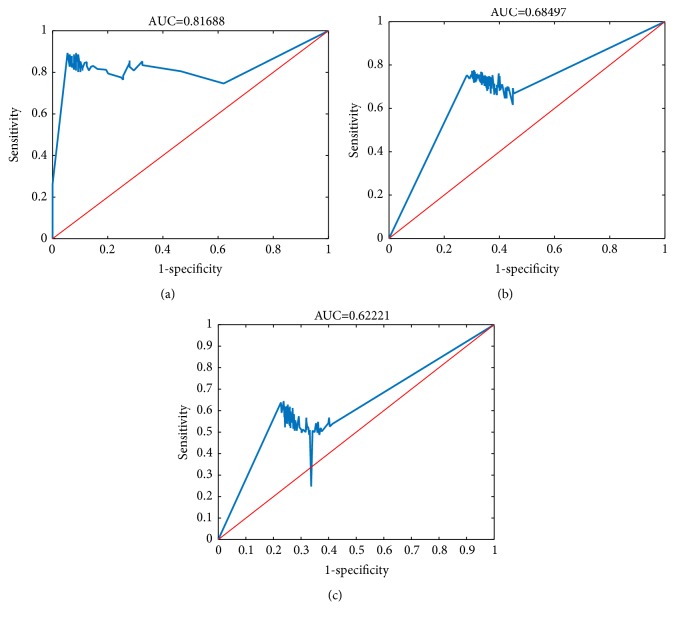
The ROC curves of the three predictors. (a) The ROC curve of the aging predictor; (b) the ROC curve of the AD predictor; and (c) the ROC curve of the network predictor.

We analyzed the gene expression data, including 11,333 genes based on 1,251 normal samples from 22 tissues and 974 AD samples from 20 tissues (Table S1). The details of samples in the training dataset and the independent test dataset were shown in Table 1. We applied the reliefF algorithm to rank the 11,333 genes and the nearest neighbor algorithm (NNA) to construct the aging and AD predictors, respectively. Then, the 5-fold cross-validation was used to evaluate the predictive accuracies and select proper number of key markers. The learning curves in the training datasets were shown in Figures 2(a) and 2(b). We considered the top 44 genes as aging related risk biomarkers and the top 98 genes as AD related risk biomarkers (Table S2), based on the cross-validation. Next, the two predictors were validated in the independent test datasets. The receiver operating characteristic (ROC) curves were shown in Figures 3(a) and 3(b). The area under the ROC curve (AUC) for the aging predictor was 0.8169 and the AD predictor was 0.6850. Furthermore, the accuracy of the aging predictor was 0.8806 and the AD predictor was 0.7359. In summary, these results showed that the predictors were with both high accuracy and efficiency.

The selected biomarkers were with critical functions. For example, among the 44 aging biomarkers, the gene with the highest ranking was USMG5 (DAPIT). As Ohsakaya S and his colleagues reported, DAPIT was associated with mitochondrial ATP synthase [16]. It played an active role in the energy metabolism of cells by maintaining the ATP synthase population in mitochondria. Further, changes in energy metabolism with age were a decisive factor of aging [17]. As we grew older, the function and integrity of the cells were gradually lost, resulting in a decrease in the total amount and efficiency of energy production. This metabolic shift would further lead to a decrease in cell function and promote the progression of aging.

In addition, among the 98 AD biomarkers, the RPUSD3 gene ranked first. The protein it encodes affected the assembly of mitochondrial ribosomes by adding a pseudouridine group to 16S rRNA [18]. Thus, the loss of RPUSD3 can result in defects in mitochondrial protein production. Cai Q et al. revealed that mitochondrial disturbance was a key factor in synaptic failure and degeneration in AD [19]. Mitochondria, an important factor for synaptic function, can buffer Ca^2+^ and maintain neurotransmitter release by providing ATP. Accordingly, the perturbation of mitochondria may lead to synaptic function change and thus contribute to AD.

Interestingly, these discoveries suggested that mitochondria seemed to act as a key bridge connecting aging and AD. As aging progressed, most metabolic activities within the cell gradually declined, including the dysfunction of mitochondria. When these changes affected the normal function of neurons in the brain, they may contribute to the pathology associated with AD.

### 2.2. The Comparison of Aging Scores between Normal Samples and AD Samples

To study the aging of brain tissues, we calculated aging scores comprising the 44 aging related risk biomarkers. As mentioned in Materials and Methods, for each sample, the aging score was defined as the Euclidean distance from its nearest young normal sample minus the Euclidean distance from its nearest old normal sample. The median and mean of aging score as well as the chronological age for different age groups were shown in Tables 2 and 3, respectively. Both in the AD samples and in the normal samples, the aging score increased roughly with the chronological age. More importantly, when the age was ≥85 years old, although the average age of AD samples was lower than that of normal samples, the aging score of AD samples was still higher than that of normal samples. The results indicated that the aging rate of brain tissues in AD patients was higher than that of normal people, consistent with the previous study about AD [10]. Additionally, we were surprised to find that, compared to the chronological age, the aging scores were more informative.

**Figure 4:**
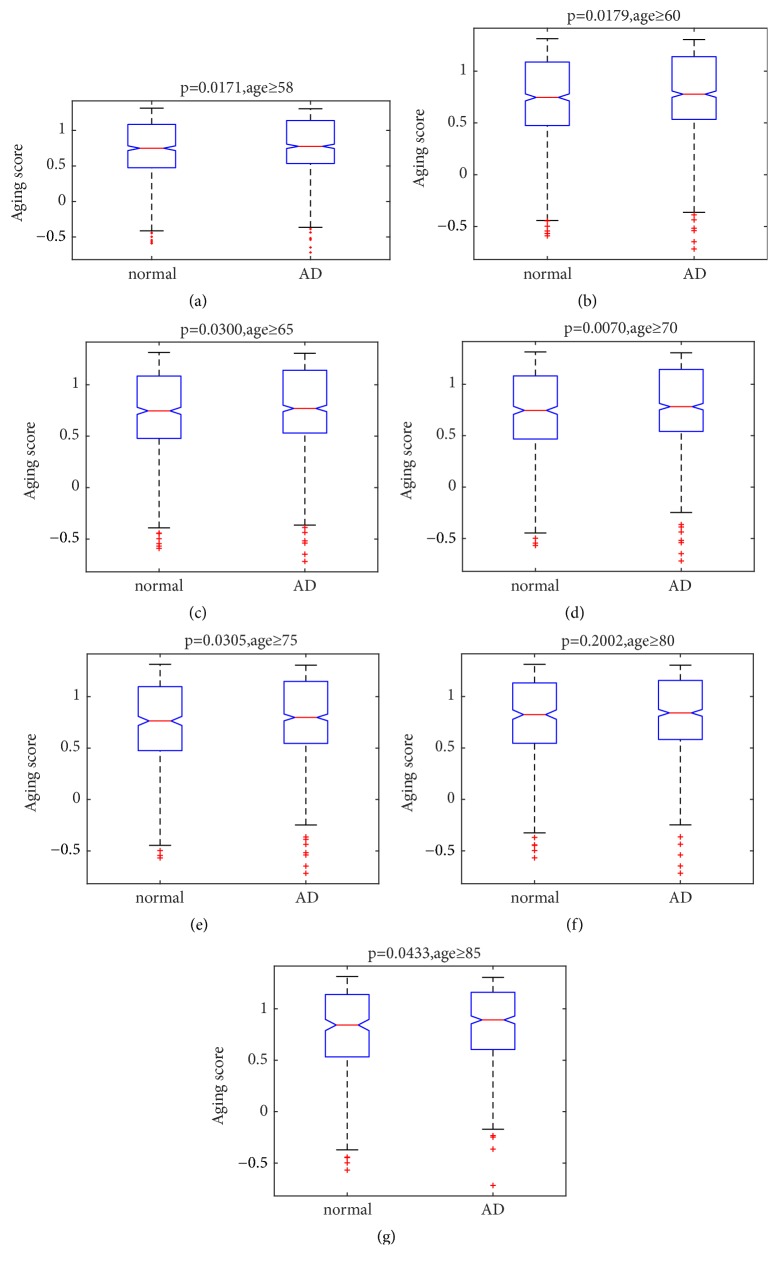
The results of the Kruskal-Wallis test for aging scores of AD samples and normal samples in different age groups. (a) Age≥58; (b) age≥60; (c) age≥65; (d) age≥70; (e) age≥75; (f) age≥80; and (g) age≥85.

Then, the Kruskal-Wallis test was performed to validate whether the aging scores reflected the difference between AD and normal samples. The test was carried out on different age groups in order to offset the effects of age, respectively. The results were shown in Figure 4. It indicated that most of the aging scores of AD samples were indeed significantly different from the normal samples (p<0.05), which implied the validity of the aging scores.

### 2.3. The Aging Acceleration Network Can Provide Insights into Key Biological Functions of AD

To better study the potential molecular mechanisms between AD and aging, we constructed an aging acceleration network using network methods (see Materials and Methods). We required p<0.05 and FDR<0.2 to ensure reliable correlation. Furthermore, the opposite sign of the correlation coefficient was also required to reflect the difference between the AD samples and the normal samples. The aging acceleration networks were constructed based on the training dataset and the test dataset (Table 1), respectively. As a result, the Fisher's exact test was carried out to verify the reliability of the aging acceleration network (p<0.05). The result showed that the two networks established with different samples were with enough similarity. Thus, it can be utilized to further analyze the relationship between aging and AD.

**Figure 5:**
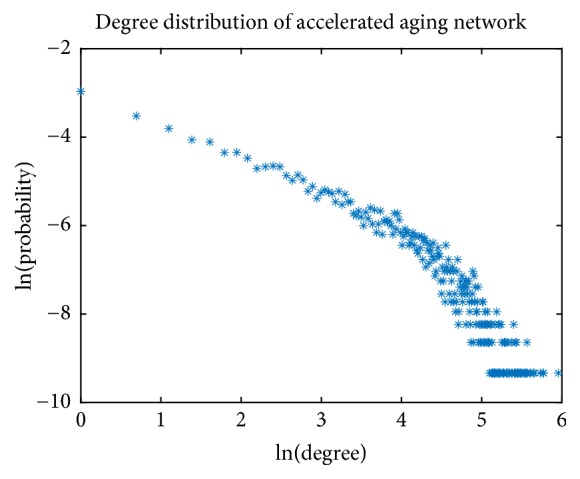
The degree distribution of the aging acceleration network.

The scale-free is one of the most important topological properties for a biological network. To verify if the network was in accordance with the scale-free characteristic, the curve of node degree distribution was drawn (Figure 5). The node degree and its corresponding probability were both logarithmically transformed. The Pearson's correlation coefficient was -0.9315 (p<0.05). It indicated that the node degree distribution was consistent with the power-law distribution. As a result, the aging acceleration network was with the important characteristic of the biological network (scale-free).

Since the nodes (genes) with higher degrees played a more dominant role in a scale-free network, we further studied genes with high degrees in the network. First, we sorted 11,333 genes by degrees in descending order. As the method of the AD predictor, NNA was used to train the model to distinguish AD samples from normal samples, and the fivefold cross-validation selected the optimal model. The learning curve was shown in Figure 2(c). The top 98 genes with high degrees can also serve as a predictor to well classify normal and AD samples (Table S2). Furthermore, it also performed very well in the independent test set——the AUC was 0.6222 and the accuracy was 0.7037. The ROC curve was shown in Figure 3(c). In addition, through the aging acceleration network, we can also understand the connections of these 98 network biomarkers. The subnetwork they formed was shown in Figure S1. Above all, it was not difficult to draw the conclusion that the network we established can indeed reflect the difference underlying AD and normal aging.

The enrichment analysis was performed on the 98 network markers to gain insights about the key biological functions of AD. The enriched KEGG pathway was neurotrophin signaling pathway (p=6.6516e-05, FDR=0.0124, shown in Figure S1). The enriched Gene Ontology (GO) Biological Processes (BP) were vacuolar transport (GO:0007034, p=4.0923e-05; FDR=0.1815).

To our knowledge, neurotrophins (NTs) belong to a family of trophic factors, including nerve growth factor (NGF), brain-derived neurotrophic factor (BDNF), neurotrophin-3 (NT-3), and neurotrophin-4/5 (NT-4/5) [20]. It played a critical role in the nervous system. NTs can support the growth and survival of neurons in healthy brains and regulate neurological functions, such as axon and dendritic growth, synapse development, and synaptic plasticity [21, 22]. Further, the entorhinal cortex (EC) was of importance to the normal cognitive function [23], which was vulnerable to AD. In the early stages of AD, the EC showed a reduction in volume [24, 25], but neurotrophins such as brain-derived neurotrophic factor (BDNF) can maintain the cortical neurons in the EC [26]. Accordingly, the dysfunction of neurotrophin signaling pathway might conduce to the development of AD.

The accumulation of toxic substances in the brain is a feature of AD. Through autophagy, cells can eliminate protein aggregates and damaged organelles to maintain their own functions. However, autophagic vacuoles (AVs) were observed to accumulate in dystrophic neurites in the AD brain [27]. This suggested that autophagy and vacuolar transport in AD patients were abnormal compared with normal brains. In a word, our results could identify critical functions of AD based on aging acceleration.

### 2.4. The Network Analysis of the Aging-AD Bipartite Graph Reflected the Relationship between Aging and AD

**Figure 6:**
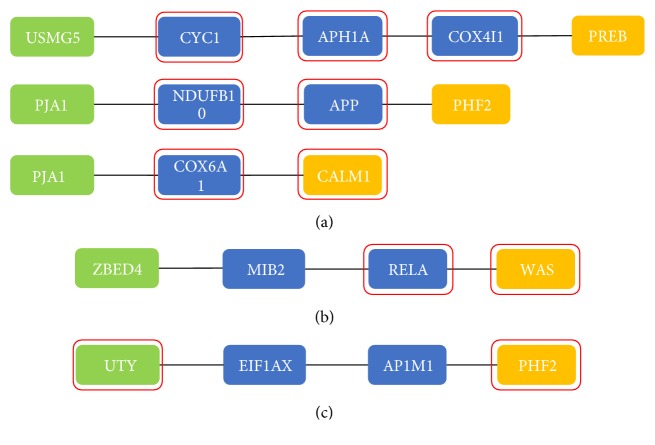
The shortest pathways (a) enriched on the KEGG pathway of Alzheimer's disease (p=1.2891e-05, FDR=0.0024; p=7.1372e-04, FDR=0.1328; p=3.5946e-04, FDR=0.0669), (b) enriched on the chemokine signaling pathway (p=9.9905e-04, FDR=0.1858), and (c) enriched on the GO BP term of protein dealkylation (GO:0008214, p=5.1337e-06, FDR=0.0228). The green nodes represented the aging biomarkers, the yellow nodes represented the AD biomarkers, and the blue nodes were the genes through which the shortest path passed. Genes that coincided with enriched functions were red-framed.

In order to dissect the relationship between AD and aging, the shortest paths between aging and AD related risk biomarkers were identified in the aging acceleration network. The enrichment analysis was then carried out on each shortest path (from each aging marker to AD marker). The most significant KEGG pathway was Alzheimer's disease (three enriched shortest paths: p=1.2891e-05, FDR=0.0024; p=7.1372e-04, FDR=0.1328; p=3.5946e-04, FDR=0.0669), the most significant GO BP term was protein dealkylation (GO:0008214, p=5.1337e-06, FDR=0.0228), and the most enriched cell signaling pathway was chemokine signaling pathway (p=9.9905e-04, FDR=0.1858). The corresponding shortest paths were shown in Figure 6.

A total of three shortest paths were enriched on the KEGG pathway of Alzheimer's disease (Figure 6(a)), and the most significant one was the pathway from USMG5 (aging marker) to PREB (disease marker). USMG5 was also the highest ranked gene in the aging predictor. As described above, USMG5 (DAPIT) affected the energy metabolism of cells by maintaining ATP synthase in mitochondria [16]. More importantly, energy metabolism was also related to AD. The brain is the most complex organ of the human body, and it requires a lot of energy to maintain its work. When energy metabolism declines, the brain is greatly affected. It has been reported that defects in glucose availability and mitochondrial function were exacerbated in AD [28]. This suggested that energy metabolism might be a factor for the progression of AD.

In addition, the most enriched GO BP term was protein dealkylation (GO:0008214). Protein dealkylation referred to the removal of an alkyl group from a protein amino acid. It has been reported that microcystin-leucine-arginine (MC-LR)-mediated demethylation of PP2Ac was associated with GSK-3*β* phosphorylation in Ser9 and contributed to the dissociation of B*α* from PP2Ac [29]. This would result in the degradation of B*α* and the disruption of the PP2A/B*α*-tau interaction, which in turn promoted tau hyperphosphorylation and paired helical filament-tau accumulation. Ultimately, these changes can lead to axonal degeneration and cell death. This indicated that protein dealkylation was associated with AD.

Furthermore, the most enriched cell signaling pathway was chemokine signaling pathway. At present, more and more studies have shown that AD can be considered as a chronic inflammation of the central nervous system (CNS) [30]. In other words, inflammatory cytokines and chemokines may play an important role in the occurrence and development of AD. It was well known that the deposition of toxic substances was one of the most prominent pathological features of AD. It was worth noting that the immunogens formed by abnormal deposition of A*β* in AD patients can lead to the activation of microglia and astrocytes (AC), which in turn led to the recruitment and release of inflammatory cytokines. Thereby, neuronal damage may be caused by direct or indirect toxic effects of the chronic immune response.

By identifying the shortest paths between aging and AD biomarkers, we can understand the connection of the two biomarker sets in the network, resulting in the formation of the aging-AD bipartite graph. Considering that biomarkers with more edges may have a more significant effect, we deeply analyzed the aging biomarker (ZNF740, SNX12, GANAB) that linked the most AD biomarkers and the AD biomarker that linked the most aging biomarkers. Of the 31 AD biomarkers with the most connections, we selected the top one, RPUSD3, for reliefF. It was also the top gene in the 98 AD biomarkers, which illustrated that it was very essential. As described above, it affected the assembly of mitochondrial ribosomes. More importantly, it has been reported that mitochondria were closely related to the function of synapses in AD [19].

Sorting nexin 12 (SNX12) is one of the PX domain-containing sorting nexin [31]. In the fetal brain, SNX12 expression increased during the embryonic stage but gradually decreased after birth [32]. One of its important functions was to inhibit the production of *β*-amyloid (A*β*) by interacting with *β*-site APP-cleaving enzyme 1 (BACE1) [33]. Zinc Finger Protein 740 (ZNF740), a protein-coding gene, was involved in transcriptional regulation [34]. Glucosidase II alpha subunit (GANAB), a protein-coding gene, can regulate hydrolase activity [34]. It was known that the deposition of A*β* was the chief culprit in AD. Acyl-peptide hydrolase (APEH) could remove excess A*β* peptide and its expression in AD brain regions rich in A*β* plaques was increased [35]. In summary, our result found the important aging biomarkers which possibly regulate AD.

**Figure 7:**
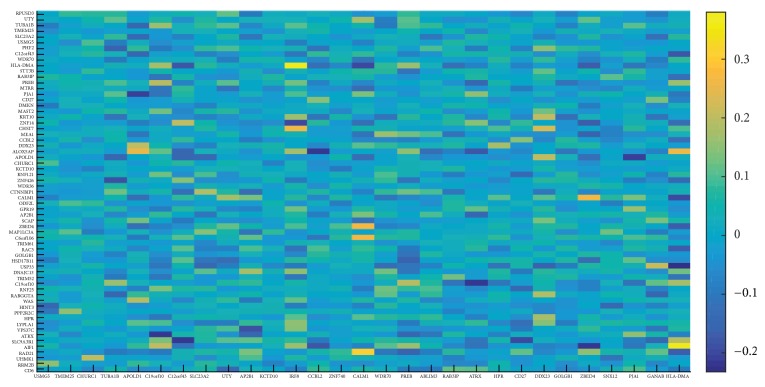
The correlation coefficients between the aging biomarkers and the AD biomarkers in AD samples. The color reflected the value of the correlation coefficient.

To further understand the correlation between aging and AD biomarkers, we also used the AD sample data in the training set for constructing network to calculate the correlation coefficients between them (Figure 7), based on the aging-AD bipartite graph. The pair of biomarkers with the highest correlation coefficient was IRF8 and HLA-DMA.

Interferon regulatory factor 8 (IRF8) was a gene with age-related expression changes, the expression of which decreased with aging [36]. In addition, IRF8 was also involved in microglial activation and neuroinflammation in AD [37]. Major histocompatibility complex, class II, DM*α* (HLA-DMA) was closely related to immunity, and its related functions included immune response NFAT and MHC class II protein complex binding [34]. Perhaps, in the accelerated aging of AD, HLA-DMA might be regulated by IRF8

Therefore, the results indicated that aging may contribute to the development of AD by affecting the function of the immune system and the energy metabolism process, where the energy metabolism may affect AD by having an impact on the immune system. Aging is a complex process involving multiple system functions. In fact, it is widely acknowledged that almost all physiological functions change with age, especially energy production [17]. Obviously, the reduction in energy production has a nonnegligible effect on the decline of the body's function. In addition, experiments have shown that the immune system played a leading role in AD [38]. For example, microglia in the brain can be used as a cleaner to phagocytose and remove debris and toxic products from the brain. The accumulation of these products in the brain was one of the pathologies of AD [39, 40]. Considering that energy decline was strongly associated with the dysfunction of the immune system [41], we believed that the immune system may be one of the most key factors for AD.

On the one hand, the total amount and efficiency of energy production gradually decreased with aging, which in turn caused the disorder of the immune system. Furthermore, it may affect the normal clearance of the immune system, leading to the accumulation of harmful substances in the brain and the occurrence of AD. On the other hand, the immune response activated by the deposition of toxic substances may cause damage to neurons, thereby promoting the development of AD.

## 3. Conclusions

In this paper, we developed an aging predictor and an AD predictor using machine learning methods, respectively. Besides, the aging score was calculated to reflect the aging state of brain tissues. It was found that most of the aging scores of AD samples were significantly higher than that of the normal samples. Furthermore, an aging acceleration network was constructed to reflect the difference between normal aging and AD. It was also confirmed that some of the hub genes in the network can be used to accurately distinguish normal samples from AD samples. In addition, the aging-AD bipartite graph was constructed to further investigate the relationship between aging and AD. Finally, through a series of enrichment analyses and network analyses, we concluded that the immune system may be one of the most critical factors for AD. Our findings supported further researches about potential relationships between AD and aging.

## 4. Materials and Methods

### 4.1. Gene Expression Profiles and Data Preprocessing

The mRNA samples were obtained from Gene Expression Omnibus (GEO) database, including GSE84422, GSE63063, and GSE15745 [42]. The sample would be chosen if (i) it was from a definite AD patient or a normal individual and (ii) the number of the samples from the same tissue was greater than or equal to ten. Since these samples were profiled on different GEO Platforms (GPLs): GPL570, GPL96, GPL97, GPL10558, GPL6947, and GPL6104, the probes from the six platforms were merged in the analyses. Then the probe sets were mapped to their corresponding gene symbols. And the expression values of the probes corresponding to the same gene were summarized. Furthermore, to ensure the reliability, genes with missing values ≥30% were deleted. In this way, we eventually collected 974 samples of AD patients from 20 different tissues and 1,251 samples of normal controls from 22 different tissues. All the samples contained 11,333 gene expression values.

For the convenience of calculation, we performed the following data preprocessing. First, these samples were transformed by the logarithmic transformation method. Then, in order to assess the sources of intersample variation, the Singular Value Decomposition (SVD) was used on the profiles from the same tissue. Finally, to eliminate the difference between gene dimensions, these profiles were normalized by the z-score method.

### 4.2. The Establishment of an Aging/AD Predictor

In order to make the model more robust, the data were divided into training dataset and test dataset. The training data set was used to train a model, and the test data set was to evaluate the performance of the model. The criteria for dividing were as follows: (i) the ratio of training dataset samples to test dataset samples was close to 2:1 and (ii) the ratio of the number of young and old samples or the ratio of AD and normal samples in the two datasets was similar. To exclude irrelevant features which could lead to overfitting, feature selection was performed using the reliefF algorithm. Considering it was time-consuming to analyze the high-dimensional gene expression data, the balance of the model complexity and predictive performance and the model efficiency, we only studied the top 100 models while training the predictor.

When developing the aging predictor, 1,251 normal samples were randomly divided (Table 1). First, samples older than 50 were labeled as 1, and those younger than 50 (including 50 years old) were labeled as 0. Then, the 11,333 genes were sorted using the reliefF algorithm and NNA was used to construct the aging predictor. The distance metric was one minus the cosine of the included angle between observations. The fivefold cross-validation was utilized to select the optimal model.

When constructing the AD predictor, the 1,251 normal samples and the 974 AD samples were together randomly divided. The AD samples were labeled as 1, and the normal samples were labeled as 0. The following computational pipeline was the same as above.

### 4.3. The Calculation of Aging Scores and the Construction of an Aging Acceleration Network

For each sample, the aging score was calculated. First, the 1,251 normal samples were divided into a young group and an old group. All samples in the young group were ≤50 years old and the samples in the old group were >50 years old. Then, for each sample, we scored the difference of the Euclidean distance from the nearest young normal sample and from the nearest old normal sample as its aging score. Finally, for the sake of analysis, the inverse tangent transformation (atan) was performed on aging scores.

In order to further dissect the relationship between aging and AD, we then constructed an aging acceleration network based on the aging scores. For the convenience of the network validation, the aging acceleration networks were constructed based on training dataset and test dataset, respectively. The pipeline of building the network was as follows. First, the product of the expression values of any two of the 11,333 genes was calculated. Then the Pearson's correlation coefficient between those products and aging scores was also calculated. The statistical significance was evaluated by p values and the Benjamini-Hochberg (BH) False Discovery Rates (FDRs). The above two steps were performed in the AD samples and the normal samples, separately. Finally, the edge between the two genes was retained if (i) p <0.05 in both AD and normal samples; (ii) FDR<0.2 in both AD and normal samples; and (iii) the sign of the correlation coefficient in the AD samples and the normal samples were different. In this way, we obtained two networks. The Fisher's exact test was carried out to verify the reliability of the aging acceleration network.

### 4.4. The Construction of the Aging-AD Bipartite Graph

In order to get more details about the relationship between aging and AD, the aging-AD bipartite graph was built as follows.

The shortest paths between the 44 aging related risk biomarkers and the 98 AD related risk biomarkers were found in the context of the aging acceleration network using the Dijkstra algorithm. This step was carried out 44*∗*98 times. If there was an existing shortest path, the connection between the aging and AD biomarkers was indicated. In this way, we can understand the connection of aging related risk biomarkers to AD related risk biomarkers in the aging acceleration network. Thus, the aging-AD bipartite graph was constructed, including 29*∗*63 aging-AD pairs (with the shortest path in the aging acceleration network).

### 4.5. Enrichment Analysis

To obtain information about the biological functions, the enrichment analysis was performed. Information of GO terms (containing all GO gene sets), GO biological processes (BP), and KEGG pathways were downloaded from Gene Set Enrichment Analysis (GSEA) (version 6.2) [43, 44]. The hypergeometric test was performed to estimate the enrichment of these selected genes compared to known pathways. The formula of the hypergeometric test is

(1) $$\begin{array}{c} p\left(\;X\geq x\;\right)=1-\overset{x-1}{\underset{k=0}{\sum }}\frac{C_{M}^{k}*C_{N-M}^{n-k}}{C_{N}^{n}} \end{array}$$

where N is the total gene number of the gene expression profiles, M is the number of known genes (i.e., GO terms or KEGG pathways), n is the number of the AD/aging biomarkers or the genes within modules, and k is the number of common entries between the known genes and the identified genes. The p value was then corrected by the FDR using Benjamini-Hochberg method. The threshold for p value was 0.05 and for FDR was 0.2.

## Figures and Tables

**Table 1:** The details of the data sets used to construct the aging predictor, the AD predictor, and the network predictor as well as the data set used to construct the aging acceleration network.

	Training data set	Test data set
The data set for the aging predictor	Age≤50: 275 samples	Age≤50: 129 samples
	Age>50: 575 samples	Age>50: 272 samples

The data set for the AD predictor, the network predictor, and the aging acceleration network	Normal: 850 samples	Normal: 401 samples
	AD: 662 samples	AD: 312 samples

**Table 2:** The mean and median aging scores of AD and normal samples from different age groups.

Age	The median aging score of AD samples	The median aging score of normal samples	The mean aging score of AD samples	The mean aging score of normal samples
≥58	0.7764	0.7497	0.7724	0.7209
≥60	0.7778	0.7468	0.7728	0.7217
≥65	0.7700	0.7465	0.7702	0.7219
≥70	0.7823	0.7461	0.7772	0.7144
≥75	0.7978	0.7640	0.7867	0.7302
≥80	0.8415	0.8246	0.8159	0.7791
≥85	0.8924	0.8426	0.8504	0.7783

**Table 3:** The mean and median age of AD and normal samples from different age groups.

Age	The median age of AD samples	The median age of normal samples	The mean age of AD samples	The mean age of normal samples
≥58	85	79	83.9928	79.3276
≥60	85	80	84.0979	79.6969
≥65	86	82	84.9914	80.7447
≥70	87	84	85.4547	83.7255
≥75	87	86	86.9070	86.9046
≥80	87	87	88.0232	88.9252
≥85	89	89	90.2199	91.1400

## Data Availability

We obtained gene expression used in this paper from the Gene Expression Omnibus (GEO, https://www.ncbi.nlm.nih.gov/geo/) database, and the series were GSE84422, GSE63063, and GSE15745.

## Supplementary Materials

Supplementary 1
* Table S1*: the details of samples from GSE84422, GSE63063, and GSE15745.

Supplementary 2
* Table S2*: the genes included in the aging predictor, the AD predictor, and the network predictor.

Supplementary 3
* Figure S1*: the subnetwork consisting of the 98 network biomarkers.
