# Supplementary material for: The Model of Aging Acceleration Network Reveals the Correlation of Alzheimer's Disease and Aging at System Level
Source: Biomed Res Int. 2019 Jul 14;2019:4273108. doi: 10.1155/2019/4273108 (PMC6662274; doi:10.1155/2019/4273108)
Supplement: Supplementary 1 — Table S1: the details of samples from GSE84422, GSE63063, and GSE15745. [file 4273108.f1.docx]

**Additional file 1.** The details of samples from GSE84422, GSE63063 and GSE15745.

The samples in GSE84422

| tissue | GPL96 | | GPL97 | | GPL570 | |
| --- | --- | --- | --- | --- | --- | --- |
|  | AD | Normal | AD | Normal | AD | Normal |
| Amygdala |  |  |  |  | 17 | 15 |
| Anterior_Cingulate | 20 | 16 | 20 | 16 |  |  |
| Caudate_Nucleus | 18 | 11 | 18 | 11 |  |  |
| Dorsolateral_Prefrontal_Cortex | 17 | 16 | 17 | 16 |  |  |
| Frontal_Pole | 24 | 15 | 24 | 15 |  |  |
| Hippocampus | 18 | 11 | 18 | 11 |  |  |
| Occipital_Visual_Cortex | 14 | 13 | 14 | 13 |  |  |
| Posterior_Cingulate_Cortex | 23 | 12 | 23 | 12 |  |  |
| Parahippocampal_Gyrus | 23 | 15 | 23 | 15 |  |  |
| Putamen | 20 |  | 20 |  |  |  |
| Precentral_Gyrus | 19 |  | 19 |  |  |  |
| Prefrontal_Cortex | 21 | 11 | 21 | 11 |  |  |
| Inferior_Temporal_Gyrus | 19 | 14 | 19 | 14 |  |  |
| Inferior_Frontal_Gyrus | 19 | 11 | 19 | 11 |  |  |
| Superior_Parietal_Lobule | 13 | 13 | 13 | 13 |  |  |
| Superior_Temporal_Gyrus | 22 | 14 | 22 | 14 |  |  |
| Nucleus_Accumbens |  |  |  |  | 17 | 13 |
| Middle_Temporal_Gyrus | 20 | 14 | 20 | 14 |  |  |
| Temporal_Pole | 18 | 14 | 18 | 14 |  |  |

The samples in GSE84422

| tissue | GPL6947 | | GPL10558 | |
| --- | --- | --- | --- | --- |
|  | AD | Normal | AD | Normal |
| blood | 145 | 104 | 139 | 135 |

The samples in GSE15745

| tissue | GPL6104 (Normal) |
| --- | --- |
| cerebellum | 146 |
| frontal_cortex | 146 |
| pons | 145 |
| temporal_cortex | 147 |
