# Supplementary material for: The Model of Aging Acceleration Network Reveals the Correlation of Alzheimer's Disease and Aging at System Level
Source: Biomed Res Int. 2019 Jul 14;2019:4273108. doi: 10.1155/2019/4273108 (PMC6662274; doi:10.1155/2019/4273108)
Supplement: Supplementary 2 — Table S2: the genes included in the aging predictor, the AD predictor, and the network predictor. [file 4273108.f2.docx]

**Additional file 2.** The genes included in the aging predictor, the AD predictor and the network predictor.

The genes included in the aging predictor.

| USMG5 | TMEM25 | CHURC1 | RPS4Y1 | TRPC4AP |
| --- | --- | --- | --- | --- |
| TUBA1B | TMSB4Y | APOLD1 | C19orf10 | SCG3 |
| C12orf43 | SLC23A2 | UTY | AP2B1 | GSTM1 |
| KCTD10 | IRF8 | CCBL2 | ZNF740 | CALM1 |
| WDR70 | BPHL | LXN | IPO7 | PREB |
| ABLIM3 | TERF2IP | UST | RAB3IP | PRPH2 |
| ATRX | HPR | IQCB1 | CD27 | DDX23 |
| GOLGB1 | ZBED4 | SNX12 | PJA1 | GANAB |
| PSMD13 | HLA-DMA | FADD | CTSF |  |

The genes included in the AD predictor.

| RPUSD3 | UTY | TUBA1B | SCG3 | CTSF |
| --- | --- | --- | --- | --- |
| TMEM25 | SLC23A2 | USMG5 | PSMD5 | PHF2 |
| C12orf43 | WDR70 | TXNIP | HLA-DMA | STT3B |
| RAB3IP | NDRG2 | PREB | BPHL | MTRR |
| PJA1 | CD27 | DLST | DMKN | MAST2 |
| UST | KRT10 | ZNF14 | RPS4Y1 | CHST7 |
| LXN | TRPC4AP | MEA1 | CCBL2 | FADD |
| DDX23 | ALOX5AP | APOLD1 | CHURC1 | KCTD10 |
| RNF121 | ZNF426 | PINX1 | WDR36 | PCDH18 |
| CLDN10 | CTNNBIP1 | CALM1 | ODF2L | GPR19 |
| AP2B1 | MRPL40 | SCAP | CYP26A1 | ZBED4 |
| MAP1LC3A | ACACA | POMZP3 | EFHB | C6orf106 |
| FXR2 | PGLYRP2 | AKR1C3 | TERF2IP | TRIM61 |
| RAC3 | GOLGB1 | PRPH2 | HSD17B11 | USP33 |
| DNAJC13 | GPC5 | TRIM52 | TICAM1 | C19orf10 |
| RNF25 | RABGGTA | WAS | HINT3 | PPP2R2C |
| HPR | FKBP2 | CAMKK2 | LYPLA1 | ENTPD4 |
| VPS37C | ATRX | NDRG4 | SLC9A3R1 | CSF1R |
| AIF1 | TMSB4Y | OLFML3 | PSMD13 | RAD21 |
| UHMK1 | RRM2B | CD6 |  |  |

The genes included in the network predictor.

| MEGF10 | CASC4 | TGOLN2 | TIMP2 | AP2A1 |
| --- | --- | --- | --- | --- |
| PAPD4 | ADIPOR2 | ITM2B | MAN2A1 | SPRYD3 |
| RBP7 | MAGED1 | SLC22A15 | LCOR | KIF3A |
| NDUFB10 | AP1M1 | AP3D1 | FAM73A | ITFG1 |
| KHDRBS1 | APBB1IP | JPH3 | MAF1 | TMCC3 |
| UBE2D3 | GNPTG | FBXO33 | RAB40C | SLAIN1 |
| ARRDC3 | CALM1 | PAIP2 | SLC25A3 | MRPL50 |
| ALKBH5 | GNAQ | SLC44A1 | COPA | L3MBTL2 |
| NT5DC1 | TMEM106B | PHF3 | SBK1 | COMMD2 |
| ZFYVE20 | SF3B1 | FAM107B | ELOF1 | HIATL1 |
| UBLCP1 | UBE2O | DPY19L4 | CCNL1 | SIN3A |
| FAM120B | NUMB | USP11 | YWHAB | SCOC |
| TMEM55A | DUSP28 | HECTD1 | VPS39 | YWHAE |
| BNIP2 | RBX1 | SNX14 | EDIL3 | PA2G4 |
| VPS4B | RFFL | OTUD6B | PSMA2 | LBR |
| RNF123 | ARPC4 | CCM2 | ARHGDIB | CCND3 |
| LAMP2 | SCYL1 | GLTP | WBP2 | COMMD6 |
| MAP1LC3A | ZNF274 | SH2D3C | CMIP | DDX5 |
| DYNC1LI2 | ZNF653 | GABRA1 | BBS1 | ATF4 |
| EIF2AK1 | NPHP3 | SSBP4 |  |  |
